# A learner corpus is born this way: From raw data to processed dataset

**DOI:** 10.1016/j.dib.2022.108527

**Published:** 2022-08-08

**Authors:** Chung Hong Danny Leung, Mei Yung Vanliza Chow, Haoyan Ge

**Affiliations:** School of Education and Languages, Hong Kong Metropolitan University, Ho Man Tin, Kowloon, Hong Kong Special Administrative Region

**Keywords:** Learner language corpus, Written data, Meta data, Data processing, ‘Regular expression’ text processing technique, Natural language toolkit

## Abstract

This data article presents the development of a learner corpus (i.e. a systematic computerized web-based repository of written texts produced by language learners) from the initial phase of the development where written assignments were collected from language learners as raw data to the critical phases where the processed text data and meta data were aligned and transformed to the web interface of the corpus. The corpus developed is called the CELL (Chinese and English Learner Language) Corpus, which comprises: i) text data containing 4.2 million English words and 18 million Chinese characters; and ii) meta data including the demographic information of the participants whose text data were collected. This article first outlines the steps for collecting the text data and meta data and then explains the processes for cleaning, annotating and tagging the text data. Discussion of the problems the research team encountered with segmentation of the Chinese text data and accuracy check of the processed datasets is also included in this article. The CELL Corpus comes with the concordance and word list features which will enable language teachers and researchers to investigate frequency, accuracy and complexity of vocabulary use in learner language. The steps and processes reported in this article will inform future development of learner language corpora of different languages.

**Specifications Table**

**Table:** 

Subject	Social Sciences, Linguistics, Education
Specific subject area	Corpus linguistics, Chinese and English learner language, Academic writing
Type of data	Text data and meta data
How the data were acquired	1. Text data The text data were collected from undergraduate students’ academic essay-type assignments in Chinese and English. The collected academic essays were then converted into text files for cleaning, anonymization, part-of-speech (POS) tagging and annotation. The accuracy of the processed text data were tested by a python script before they were uploaded to the web-based corpus using the In-Memory Processing Architecture with Regular Expressions. 2. Meta data The meta data were the demographic information of the undergraduate students whose academic essays were collected as the text data. Qualtrics surveys were administered to the students – to collect their demographic information, including age, gender, place of birth, first language and public examination results of English language and Chinese language.
Data format	Raw and processed
Description of data collection	1. Text data English academic essay-type assignments written by undergraduate students whose second language is English and who were majoring in language and/or education between 2018 and 2020 were collected. Chinese academic essay-type assignments written by undergraduate students whose first language is Chinese and who were majoring in language and/or education between 2018 and 2020 were collected. 2. Meta data Qualtrics surveys on the required demographic information (e.g. age, gender, etc.) were administered to the undergraduate students whose English and Chinese academic essays were collected. 3. Consent to provision of text data and meta data Return of the Qualtrics surveys was treated as a consent to both the provision of the demographic information as meta data and the provision of the collected academic essays as text data. In other words, only the academic essays written by students who returned the Qualtrics surveys were processed as text data.
Data source location	Institution: School of Education and Languages, Hong Kong Metropolitan University City/Town/Region: The Hong Kong SAR Country: China
Data accessibility	Raw data repository: DOI: 10.17632/gs4ppd7sz3.1 Mendeley data repository: https://data.mendeley.com/datasets/gs4ppd7sz3 The Chinese and English Learner Language (CELL) Corpus website: https://cellcorpusouhk.com/

## Value of the Data

• The corpus data can be useful for linguists, educators, language learners and other social scientists working on the use of Chinese and English by bilingual learners.
• These data can help researchers address questions related to second language acquisition, bilingual language education, academic writing, and intercultural communication.
• The data can be useful to educators of English and Chinese to develop teaching materials and course curriculum.
• These datasets can be used for training machine learning models to identify errors in Chinese and English learner language corpora.
• These datasets can inform future development of learner language corpora of different languages.

## 1 Data Description

The **C**hinese and **E**nglish **L**earner **L**anguage Corpus (referred to as ‘the CELL Corpus’ hereafter) is designed as a learner language corpus. A corpus is a collection of texts, utterances or other specimens considered more or less representative of a language, and usually stored as an electronic database [1], [2]. A learner language corpus is a collection of language data produced by L2 learners [3]. The CELL Corpus, as a learner language corpus, is thus designed as a collection of text data chiefly composed of Chinese and English academic essay-type assignments written by university undergraduate students. The students submitted their academic essays as assignments for the assessment purpose of the courses they enrolled for, which suggests the authenticity of the text data collected. In addition to the text data, the CELL Corpus is also designed to include the meta data of the students whose academic essays were collected. The meta data collected represent five types of demographic information of the students, which are namely: age, gender, place of birth, first language and public examination results for Chinese Language and English Language. The two datasets (i.e. text data and meta data) of the CELL Corpus are delineated in the following sub-sections.

### 1.1 Text data

The text data of the CELL Corpus contain 8129 Chinese and 2732 English academic essay-type assignments, which add up to a total of 18,029,899 Chinese characters and 4,186,653 English words. The academic essays were collected from a total of 988 university students who were majoring in one of the undergraduate programmes in the School of Education and Languages at the Open University of Hong Kong between 2018 and 2020^1^. Based on the courses the students submitted their academic essays to, the text data in the CELL Corpus are of the following six categories:

a. English/Chinese Language Education
b. English/Chinese Language Studies
c. Education Theories
d. Early Childhood Education
e. General Education

The text data are part-of-speech (POS) tagged (i.e.TagAnt) [4] and annotated according to the four sections commonly found in the academic essay genre, which are namely: Title, Introduction, Body, and Conclusion.

### 1.2 Meta data

Qualtrics surveys were administered to the undergraduate students whose academic essays were collected as text data for the CELL Corpus (see Appendix A in the Mendeley data repository for a sample of the survey). The survey aimed at collecting the students’ demographic information as meta data for the CELL Corpus. Regarding the overall design of the CELL Corpus as a learner language corpus, students’ information such as age, gender, place of birth, first language and public examination results for Chinese Language and English Language was collected as meta data.

### 1.3 Corpus features

The text data and meta data described in the above sub-sections serve as the backend datasets. The frontend of the CELL Corpus is a user-friendly website (cellcorpusouhk.com) comprising a concordance feature which produces key-word-in-context (KWIC) concordance lines, and a word list feature which shows English word/Chinese character frequency tabulations in either the ascending or descending order. There is a filter function made available by the two backend datasets. In terms of the text data, the filter function enables users to select for each search which categories of the six courses (e.g. English/Chinese Language Studies, Education Theories) and which of the four sections of academic essay (e.g. Introduction, Body) to look at. In terms of the meta data, users can search for only the text data matching a particular grouping of demographic information (e.g. male + Chinese as first language + Level 5 as English Language public exam result). By doing so, the CELL Corpus has aligned the text data with the meta data in the backend in a way that users in the frontend can make use of the filter function to look for specific data in a search (see Appendix B in the Mendeley data repository for a demonstration of the filter function with the ‘education’ concordance lines).

## 2 Experimental Design, Materials and Methods

Methods and steps adopted to process and convert the assignments, meanwhile aligning with the meta data as available data for the web-based CELL Corpus are divided into three phases, namely ‘processing text data’, ‘collecting meta data’ and ‘aligning and transforming the two sets of data onto the web interface of the CELL Corpus’. In the following sub-sections, these stages are explained in detail.

### 2.1 Processing text data

In the following sub-sections, steps taken to process the English and Chinese assignments are explained.

### 2.2 Processing English text data

**Fig. 1:**
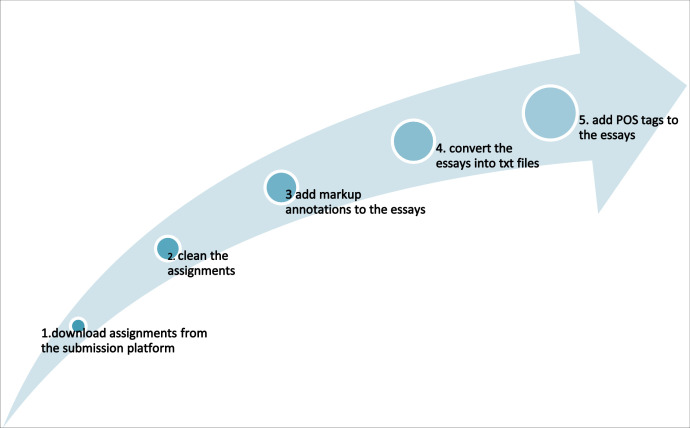
Steps taken to process the English assignments.

As shown in Fig. 1, five main steps were taken to process the English assignments.

Before downloading the students’ assignments from the assignment submission e-platform, emails were sent to them to obtain their consent. A standardised folder structure was also set up to save the anonymised student assignments into different folders. It was important to create a new layer of folder whenever a new step was conducted. This helps to ensure that all work conducted in each of the five steps was recorded.

After all the assignments were downloaded and saved into different folders, information not relating to the essay itself such as page number, cover page of the assignments, header and footer were removed (Fig. 1, Step 2), so that the CELL Corpus will enhance language studies focusing on the four main sections of an academic essay. That is, the Title, Introduction, Body Paragraphs and Conclusion. As shown in Fig. 1, these four markup annotations about mark-ups of these four sections (Step 3) and parts of speech (POS) taggers (Step 5)^2^ were added to each of the cleaned assignments to enhance researchers to focus on investigating language use in a particular section or grammatical pattern.

### 2.3 Processing Chinese text data

**Fig. 2:**
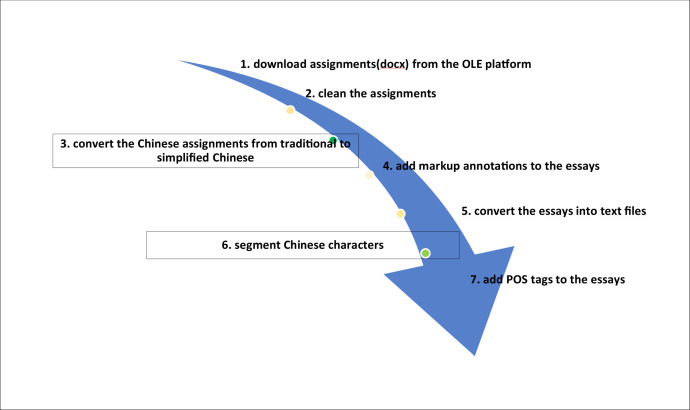
Steps taken to process the Chinese assignments.

Unlike the English language, no spacing is necessary between characters in the Chinese language. Therefore, word segmentation was conducted to segment the Chinese assignments text data. As shown in Fig.2, Steps 3 and 6 were added while processing Chinese assignments. SegmentAnt [5] which mainly draws on Jieba engine and Chinese Lexical Analysis System (ICTCLAS) developed by the Institute of Computing Technology (http://ictclas.org) was employed to segment the Chinese assignments. A pilot study was conducted to test the accuracy of the software selected. Twenty Chinese assignments were segmented with SegmentAnt and it was observed that the software displayed much higher accuracy rate in segmenting simplified Chinese^3^, comparable with the near 98% accuracy rate reported on the ICTCLAS website. The higher accuracy rate in segmenting simplified Chinese text was expected since ICTCLAS was developed by the Institute of Computing Technology in the mainland of China where simplified Chinese is used. Therefore, Step 3 was incorporated to convert all the Chinese assignments from traditional Chinese into simplified Chinese to ensure a high accuracy rate in segmenting the Chinese texts into meaning lexical units.

Although errors in segmentation may occur since the accuracy rate of the software is not 100% as stated on the website, a 98% accuracy rate is good enough to facilitate the kinds of studies this learner language corpus aims at. During the segmentation process, it was noted that grammatical errors or typing mistakes made by the students might affect the accuracy of SegmentAnt. For instance, some students might have kept an unnecessary space between Chinese characters that originally should not be separated by a space. This in turn affected the accuracy in SegmentAnt. Since it is a learner language corpus, students’ mistakes such as these were not amended to preserve their writing styles. At the time of writing the paper, manual detection and revision of wrong segmentation continue, in an attempt to further enhance the accuracy rate in segmenting Chinese text data and to use the learner language data as a tool to explore issues related to training machine learning models for segmentation.

### 2.4 Collecting meta data

As previously explained, meta data of the students were collected through the administration of Qualtrics survey (Please refer to Appendix A in the Mendeley data repository for the survey form). The survey served three functions. It helped to obtain the consent from the students; to collect backgrounds of the students, which was later transformed into search filters on the CELL Corpus website and; to screen the English and Chinese assignments from students who do not speak the English and Chinese languages as L2 and L1 respectively.

### 2.5 Aligning and transforming text data and meta data onto the web interface of the CELL Corpus

A python script was written to test the accuracy of the processed text data before uploading it on the web interface. The web interface for the CELL Corpus was developed based on Django-Python web framework in java. The natural language toolkit (NLTK) (version 3.4) of the Python package, React Typescript and Tailwind-CSS were utilised to transform the tagged and segmented assignment data (in .txt format) in a well curated standardised format on the web interface of the CELL Corpus so that users are enabled to make different kinds of search with or without applying filters. With highly customised responsive web design facilitated by Tailwind-CSS, the CELL Corpus is a single paged application that provides both desktop and mobile views.

The tools also enable search words to be displayed in key words in context format (KWIC), sort the concordance lines of the search words in vertical alphabetical orders with a default window size of 80 characters and mark the three collocations on the left and right of the search words in different colours. Frequencies of each of the word types observed in the data were also calculated and displayed through the deployment of these tools.

To facilitate text string searching, ‘in-memory processing with regular expressions’ was adopted. This processing technique of running computer calculations, namely the text search in the CELL Corpus, was conducted entirely in the computer memory itself (e.g. in RAM). This means the technique is free of cost. It simplified the technical design and architecture of the web interface for the CELL Corpus. The website developer was allowed to modify the functions of the web interface, depending on how we wanted to perform the computation in the computer memory.

The ‘regular expression’ text processing technique was incorporated in this program to offer more advanced search capability of the Corpus [6]. It enables dynamic concordance search of a sequence of characters as a search pattern in the Corpus. That is, allowing any type of text string searching we expect the Corpus to perform. When a search of a text string is requested, the search is computed from the entire CELL Corpus, displaying the text search results as concordance lines in KWIC format at a fast speed.

## Ethics Statements

The voluntary, fully informed consent of the participants involved in this data collection was obtained. The study was approved by the ethics committee and conducted in accordance with the ethical standards at Hong Kong Metropolitan University (HE29May2017–RGC2017/14).

## CRediT authorship contribution statement

**Chung Hong Danny Leung:** Conceptualization, Methodology, Investigation, Writing – original draft, Project administration, Funding acquisition. **Mei Yung Vanliza Chow:** Conceptualization, Methodology, Investigation, Data curation, Writing – original draft, Project administration. **Haoyan Ge:** Methodology, Investigation, Data curation, Writing – original draft.

## Declaration of Competing Interest

The authors declare that they have no known competing financial interests or personal relationships that could have appeared to influence the work reported in this paper.

## Data Availability

The Chinese and English Learner Language Corpus (CELL Corpus) (Original data) (Mendeley Data).

## Footnotes

1 The Open University of Hong Kong was retitled as Hong Kong Metropolitan University on 1 September 2021.

2 For the lists of English and Chinese tree taggers, please refers to the following websites: https://wenku.baidu.com/view/0e223f5d7275a417866fb84ae45c3b3567ecddd4.html?re=viewhttps://courses.washington.edu/hypertxt/csar-v02/penntable.html

3 The accuracy rate of the segmentation tool employed for the Chinese data is reported to be 98.23% on the ICTCLAS website (http://ictclas.nlpir.org/nlpir/html/fenci-1.html).
